# An Elderly Man Suffers a Renal Cell Carcinoma Metastasis in the Pineal Gland: Lessons Learned and Ethical Considerations

**DOI:** 10.7759/cureus.14771

**Published:** 2021-04-30

**Authors:** Amira M Alolyani, Ibrahim Al Luwimi, Ahmed Ammar

**Affiliations:** 1 Department of Neurosurgery, Imam Abdulrahman Bin Faisal University, King Fahd Hospital of the University, Khobar, SAU

**Keywords:** pineal gland, metastasis, renal cell carcinoma, inconclusive biopsy, bioethics, endoscopic third ventriculostomy

## Abstract

Metastases to the pineal gland are rare and reported cases have consisted mainly of lung and gastrointestinal primary malignancies. Here we report the third case in the literature of pineal gland metastasis from renal cell carcinoma.

A 69-year-old man, status post excision of right renal cell carcinoma 20 years ago, presented with a one-month history of urinary incontinence. Images revealed a solitary mass in the pineal region with obstructive hydrocephalus. Endoscopic third ventriculostomy (ETV) and biopsy of pineal mass were performed. The histological diagnosis of the biopsy was inconclusive. The patient was scheduled for a follow-up and readmission for a repeat biopsy, however, was lost to follow-up. No attempts were made by the hospital team or patient relations department to contact him. Eventually, the patient presented after 18 months to the emergency room (ER) with confusion, forgetfulness, gait disturbance, weakness of lower extremities, and vision loss due to enlarged pineal mass. Another ETV and biopsy were performed. The histological findings were compatible with metastasis from renal cell carcinoma. The patient died after three months due to rapid general deterioration in his condition.

The lessons that have been learned from this case are: 1) Metastatic tumor should be considered in the differential diagnosis of pineal region tumors, particularly in elderly patients and with a known history of malignancy; 2) If the first biopsy is inconclusive, a rapid plan and a strict follow-up for a repeat biopsy should be made; 3) Elderly patients should have special care; they should be well informed about their condition and should be contacted regularly to ensure that they receive the optimal management plan.

## Introduction

Pineal gland tumors account for 1% to 3% of all primary intracranial tumors [1]. Metastasis to the pineal gland is very rare, occurring in 0.4% to 3.8% of all metastatic intracranial tumors. Most of these metastases were incidental and diagnosed posthumously [2]. According to a previous review, the most common site of primary origin is lung carcinoma [3]. Other malignancies that have documented cases of metastases to the pineal include thyroid, breast, esophagus, pancreas, liver, stomach, colon, kidney, bladder, prostate, melanoma, and myeloma [3-13]. Renal cell carcinoma metastatic to the pineal gland has only been reported twice in the literature [10,14]. Here, we report the third case in the literature of pineal gland metastasis from renal cell carcinoma. In this report, we aim to draw attention to the importance of considering metastatic involvement in a patient with advanced age and a known malignancy. We will also discuss how the inconclusive biopsy and the delay of diagnosis could make the management challenging, particularly from an ethical point of view. 

## Case presentation

**Figure 1:**
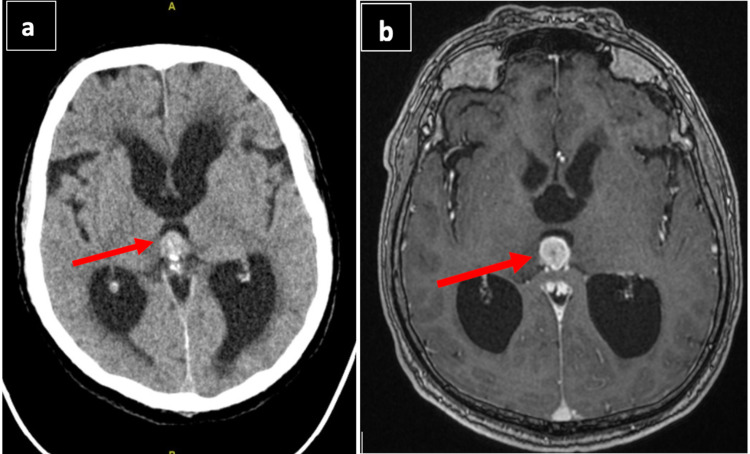
a) Plain CT scan of the brain axial view showing a hyperdense mass in the pineal region obstructing the posterior third ventricle with active hydrocephalus (arrow). b) MRI of the brain with contrast showing the same pineal region mass, hyperintense in looks, measuring 1.6x1.3x1.6 cm obstructing the posterior third ventricle with active hydrocephalus (arrow).

The patient reported in this case is a 69-year-old Saudi man, status post excision of right renal cell carcinoma 20 years ago, presented to King Fahd University Hospital on March 17, 2016, complaining of urinary incontinence for one month. On examination, the patient was conscious, alert with no systemic or neurological deficits. Brain magnetic resonance imaging (MRI) and computed tomography (CT) scan with and without contrast showed solitary pineal region mass lesion measuring 1.6x1.3x1.6 cm, occupying the posterior part of the third ventricle and causing obstructive hydrocephalus. The mass was hyperdense on plain CT scan (Figure 1a) and hyperintense with contrast on MRI (Figure 1b). Tumor markers for primary pineal tumors were negative. CT scan of chest and abdomen were normal. 

The patient was treated with endoscopic third ventriculostomy (ETV) and biopsy of the pineal mass. Hydrocephalus resolved; however, the histological diagnosis of the biopsy was inconclusive and consisted of fibrous tissue. The patient was then discharged in a stable condition and was scheduled for a follow-up appointment in the neurosurgical outpatient clinic. Readmission was planned for further management and repeated biopsy of the mass lesion. The patient did not show up in the outpatient clinic.

**Figure 2:**
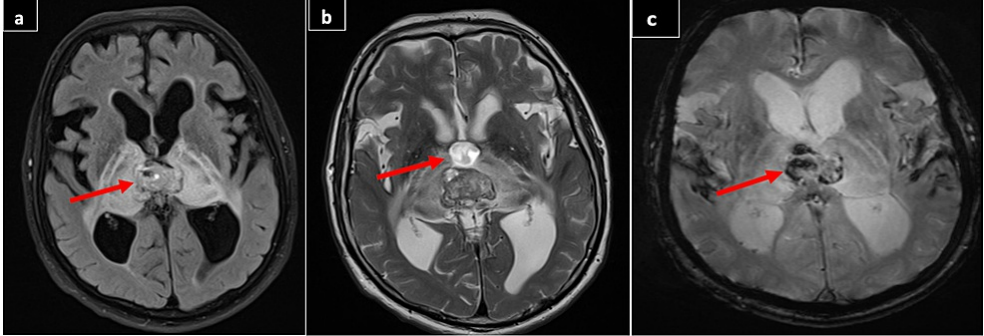
a) MRI of the brain with contrast axial view showed increased in the size of previous pineal region mass 3x2x3.5 cm with marked contrast enhancement and extensive edema (arrow). The mass and edema are extending bilaterally with thalamic compression, marked posterior third ventricular obstruction and marked active obstructive hydrocephalus. b) MRI T2 weighted image showing the same mass with heterogenous intensity (arrow). c) MRI of the brain flair view showing the same mass heterogenous signal intensity with multiple foci of low signal indicating hemorrhagic component (arrow).

One and a half years later on July 25, 2017, he presented to the ER complaining of confusion, forgetfulness, postural imbalance, difficulty in walking, and decreased vision for two weeks. On examination, he was lethargic and confused with papilledema. Brain MRI with contrast (Figure 2) showed the tumor was markedly enlarged in the pineal region, causing obstructive hydrocephalus, heterogeneous in appearance, and foci of hemorrhage. The pineal mass was extending down with mid-brain compression and bilateral thalamic compression.

**Figure 3:**
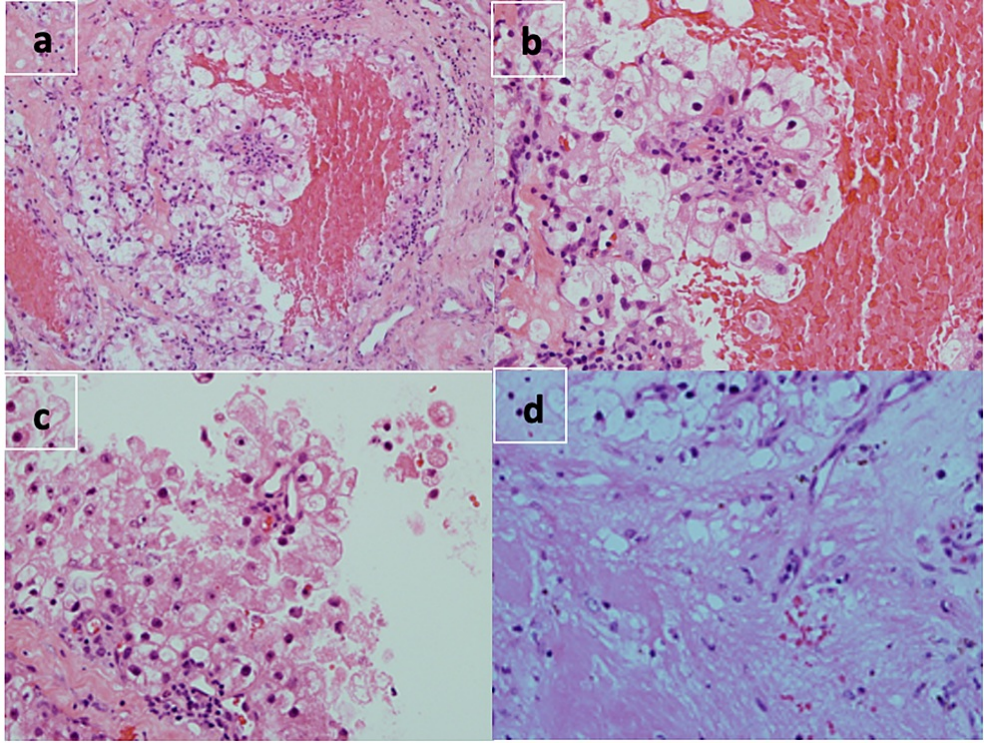
a) H&E medium power; the tumor composed of sheets of cells with clear and granular eosinophilic cytoplasm. b) H&E high power; the tumor cells exhibit mild to moderate nuclear pleomorphism with prominent nucleoli. c) H&E high power; another focus of the tumor. d) H&E high power; another focus of the tumor with glial tissue.

The patient was re-operated with an ETV and tumor biopsy. External ventricular drain (EVD) was left in the ventricle. The histological studies with hematoxylin and eosin (H&E) stain showed sheets of cells with pleomorphism that were consistent with a tumor (Figure 3).

**Figure 4:**
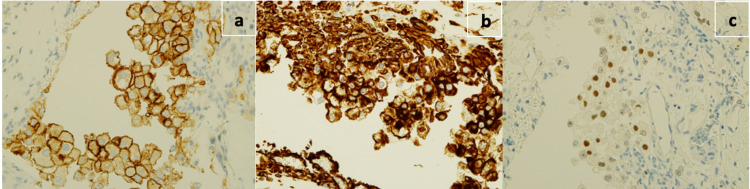
a) CD10 strong membranous staining. b) Vimentin strongly positive. c) PAX-8 strongly positive.

Immunoassay studies with the vimentin, CD10, and PAX-8 stain were also done because of the patient’s past history of renal tumor excision. The findings were positive and compatible with metastasis from renal cell carcinoma (Figure 4).

Post-operatively, the patient remained confused with the Glasgow coma scale of 13. The patient developed *Pseudomonas* in the cerebrospinal fluid (CSF), for which he received the appropriate antibiotics and it resolved within a few weeks. He had several trials of weaning from EVD but failed, so a ventriculoperitoneal (VP) shunt was inserted. The patient did not improve and on October 20, 2017, he suffered cardiorespiratory arrest and passed away.

## Discussion

Pineal metastases are exceedingly rare and considered an incidental finding that occurs late in the course of widely metastatic systemic cancer [11]. This is the third report of pineal gland metastasis from renal cell carcinoma. All tumors in the pineal region present similarly because of the involvement of neighboring brain structures. The most common presentation is headache, nausea, vomiting, and vision loss due to compression of the aqueduct of Sylvius, resulting in obstructive hydrocephalus and elevated intracranial pressure. Less common presentations include encephalopathy, Parinaud syndrome, focal weakness, or cranial nerve symptoms [15]. In this case, the patient presented with symptoms of increased intracranial pressure, and upon the second admission, he experienced encephalopathy in the form of confusion and memory impairment and also had focal weakness.

The mechanism underlying the solitary development of pineal metastasis is still not well understood. It is thought to be related to hematogenous spread due to the absence of a blood-brain barrier in the pineal region. Ortega et al. suggested that metastasis to the pineal region is mainly due to tumor cells entering the pineal region through the posterior choroidal arteries [16]. Kashiwagi et al. believed that the pineal region, which is full of numerous sinusoidal vessels without perivascular glial sheets, is more susceptible to circulating tumor cells in the blood [17].

The preoperative diagnosis of metastatic pineal tumors based on CT or MRI is often difficult. The differential diagnosis would include the diverse histological types of primary pineal tumors; however, metastasis should also be considered, especially in elderly patients and those with a history of malignancy [18]. Brain metastasis in patients with metastatic renal cell carcinoma indicates an advanced stage (stage 4) and a poor prognosis [3]. The treatment of pineal region metastases varies according to systemic conditions, histopathology, and neurological symptoms [19]. As a therapeutic strategy in this case, we performed endoscopic third ventriculostomy and biopsy of the pineal mass. We chose this approach for three main reasons: 1) CSF diversion for the treatment of hydrocephalus; 2) histopathologic identification of the pineal tumor, and 3) well tolerated by elderly and unstable patients.

In the first admission, the histopathology report was inconclusive, and the plan was for elective admission for a repeat biopsy. However, the patient did not observe the follow-up plan and presented late after 18 months with deterioration in his condition due to an enlarged pineal mass. Another endoscopic third ventriculostomy and biopsy was performed. The positive histopathological staining with vimentin, CD10, and PAX-8 proved the source of metastasis from renal cell carcinoma. These staining and others such as RCC antigen, CA9, Pax 2 are usually positive for renal cell carcinoma [20]. 

This particular event raises important ethical concerns on dealing with a patient who did not follow the instructions of the treatment team. Understanding and respecting patient’s rights is crucial in our daily practice. The ethical code of the American Medical Association included nine principles of medical ethics as the main component of the Ethical Code. These principles are: 1) Ethics of patient-physician relationship, 2) Ethics of consent, communication & decision making, 3) Ethics of privacy, confidentiality, and medical records, 4) Ethics of genetics and reproductive medicine, 5) Ethics of caring for patients at the end of life, 6) Ethics of organ procurement and transportation, 7) Ethics of medical research and innovation, 8) Ethics of physicians and health care community, and 9) Ethics of interprofessional relationship [21]. The concept of Values-Based Medicine (VsBM) which was introduced recently focused on the theory that “the patient is the center of care” and describes the ethical principles needed in order to implement the ethical principles in every step of the daily practice. These ethical principles are: autonomy, beneﬁcence, non-maleﬁcence, justice, dignity, truthfulness, and honesty [21].

The patient has the right to be informed about the seriousness of the disease and the consequences of refusal of treatment or neglecting the follow-up plan [21]. For example, patients who were operated on and their histopathology results were inconclusive, should be informed that the risk of tumor is still there, and they must go for a second surgery for a biopsy and possible adjuvant therapy. Patients also have a duty to themselves to advocate on behalf of their own rights. If the patient is confused or doesn’t have the mental capacity to make decisions on their own, then their family members are allowed to participate and be informed about the details of the management plan. Moreover, patients have the right to continuity of care, including access to outpatient clinics and adequate follow-up after discharge from the hospital. For instance, the coordinators of the outpatient clinics should receive a list of patients who may have serious conditions and should ensure that these patients have the priority to be seen earlier. If the received treatment was not properly executed, or there have been medical mistakes or errors, the patient and the family should have the right to complain to the hospital authorities (e.g., the public relations department or to an administrator) [21]. Medical errors are more common in surgical specialties. A surgeon should take the lead in disclosing errors to patients and their families. Disclosure will promote public trust as well as justice and will also prevent further harm. Moreover, disclosure respects the patient and his/her autonomy [21].

## Conclusions

The lessons that have been learned from this case are: 1) Metastatic tumor should be considered in the differential diagnosis of pineal region tumors, particularly in elderly patients and with a known history of malignancy, 2) If the first biopsy is inconclusive, a rapid plan and a strict follow-up for a repeat biopsy should be considered, and 3) Elderly patients should have special care and should be well informed about their condition and be contacted regularly to ensure that they receive the optimal management plan.
